# Longitudinal monitoring of *Culicoides* in Belgium between 2007 and 2011: local variation in population dynamics parameters warrant cautious use of monitoring data

**DOI:** 10.1186/s13071-018-3082-3

**Published:** 2018-09-17

**Authors:** Charlotte Sohier, Isra Deblauwe, Reginald De Deken, Maxime Madder, Christiane Fassotte, Bertrand Losson, Nick De Regge

**Affiliations:** 1 Enzootic, vector-borne and bee diseases, Sciensano, Brussels, Belgium; 2 Department of Biomedical Sciences, Institute of Tropical Medicine, Antwerp, Belgium; 3 Department of Veterinary Tropical Diseases, University of Pretoria, Pretoria, South Africa; 4 Plant Protection and Ecotoxicology, Life Science Department, Walloon Agricultural Research Centre (CRA-W), Gembloux, Belgium; 5 Department of Infectious and Parasitic Diseases, Faculty of Veterinary Medicine, University of Liège, Liège, Belgium

**Keywords:** *Culicoides*, Risk assessments, Entomological monitoring, Vector-free period, Belgium

## Abstract

**Background:**

Several European countries suffered important economic losses during the past decade due to the emergence of bluetongue and Schmallenberg viruses. Both are viruses of veterinary importance and are spread by *Culicoides* spp. This triggered many European countries to start *Culicoides* population monitoring. Recently a one year monitoring study at 16 sites in Belgium revealed that important variation existed in *Culicoides* abundance and species diversity between collection sites. In order to analyze whether this variation is consistent over years, a detailed analysis of monitoring data collected at seven locations in Belgium between 2007 and 2011 was performed in this study. At all locations, biting midges were collected with OVI black light traps set-up in close proximity to livestock.

**Results:**

In total, 42 different *Culicoides* species were morphologically identified. Species of the subgenus *Avaritia* represented 83% of all collected midges. Nevertheless, important differences in species composition were found between sites. Furthermore, statistical differences between sites were found for the total and maximum annual abundance, showing that a consistent higher or lower number of *Culicoides* could be collected depending on the selected collection site. Yearly, up to 16 and 30-fold differences in total and maximum annual abundances between sites, respectively, were found. Also the month in which most *Culicoides* were collected varied greatly between years, both at local (from May to October) and country level [May (2008), June (2010), July (2009), August (2011), October (2007)]. Finally, the average vector-free period over all sites and years was 173 days and could roughly be defined between November and the end of April. Interestingly, important yearly variations of up to two months in the duration of the vector-free period were found between the studied collection sites. In contrast to the abundance parameters, no specific sites could however be identified where monitoring consistently showed shorter or longer vector-free periods.

**Conclusions:**

In conclusion, our results show that the selection of collection sites for *Culicoides* monitoring, even in a small country such as Belgium, strongly influences abundance parameters and that yearly variation in seasonality occurs. This emphasizes that care should be taken when using such parameters in risk assessments for transmission of *Culicoides*-borne diseases and that more clear and strict guidelines for *Culicoides* monitoring should be considered when monitoring data are used for legislative purposes.

**Electronic supplementary material:**

The online version of this article (10.1186/s13071-018-3082-3) contains supplementary material, which is available to authorized users.

## Background

*Culicoides* (Order Diptera, Family Ceratopogonidae) are small hematophagous insects distributed worldwide. They are known to transmit numerous pathogens, including bluetongue virus (BTV) and Schmallenberg virus. These viruses have caused considerable economic losses for European farmers and livestock industry during the past decade. Commission Regulation (EC) No 1266/2007 [1] introduced the obligation for the Member States to carry out bluetongue monitoring and surveillance programmes aimed at detecting any possible incursions of the bluetongue virus, demonstrating the absence of certain serotypes (when appropriate) or determining the seasonally vector-free period through entomological surveillance.

These monitoring studies, mostly using Onderstepoort Veterinary Institute blacklight traps (OVI traps), provided information on species composition, abundance and seasonality of these vectors in different countries. They also highlighted the importance of temperature, humidity and climate in general as important drivers for these parameters [2–5].

Besides the usefulness of monitoring to understand *Culicoides* population dynamics, abundance data might be interesting to assess the risk of disease transmission in specific regions. Viennet et al. [6, 7] showed that UV-light trap collections were linearly correlated to attack rates on animals for several *Culicoides* species. Within VectorNet, a currently ongoing project of EFSA and ECDC, efforts are undertaken to obtain data that could be used to produce abundance maps for the putative *Culicoides* vector species [5], which could later on be used for risk assessment.

*Culicoides* monitoring data are currently already used to regulate animal transport in the specific case of a bluetongue virus outbreak. In response to the bluetongue virus serotype 8 virus (BTV-8) outbreak in Europe in 2007, the European Union adopted different control measures on restriction in movement of animals and on abundance of vectors [1]. A seasonally vector-free period was defined, in order to allow the movement of animals if specific criteria are met. The vector-free period is defined as the period in which less than five parous *Culicoides* per trap are collected and *C. imicola* is completely absent. An overview of seasonally vector-free periods reported by different countries can be found online [8].

The examples described above show the importance of collecting representative *Culicoides* monitoring data. Interestingly, a detailed analysis of a one year monitoring study in 2011 at 16 collection sites distributed over four regions in Belgium has shown a high variability in *Culicoides* species abundance, seasonality and species diversity at individual, even nearby, collection sites in Belgium [3]. This indicated that the selection of a collection site could strongly influence parameters describing population dynamics. Here we perform a detailed analysis of *Culicoides* monitoring data gathered in Belgium during a five year period to study whether such an observed variation among collection sites is consistent over years.

## Methods

### *Culicoides* trapping and collection sites

**Fig. 1:**
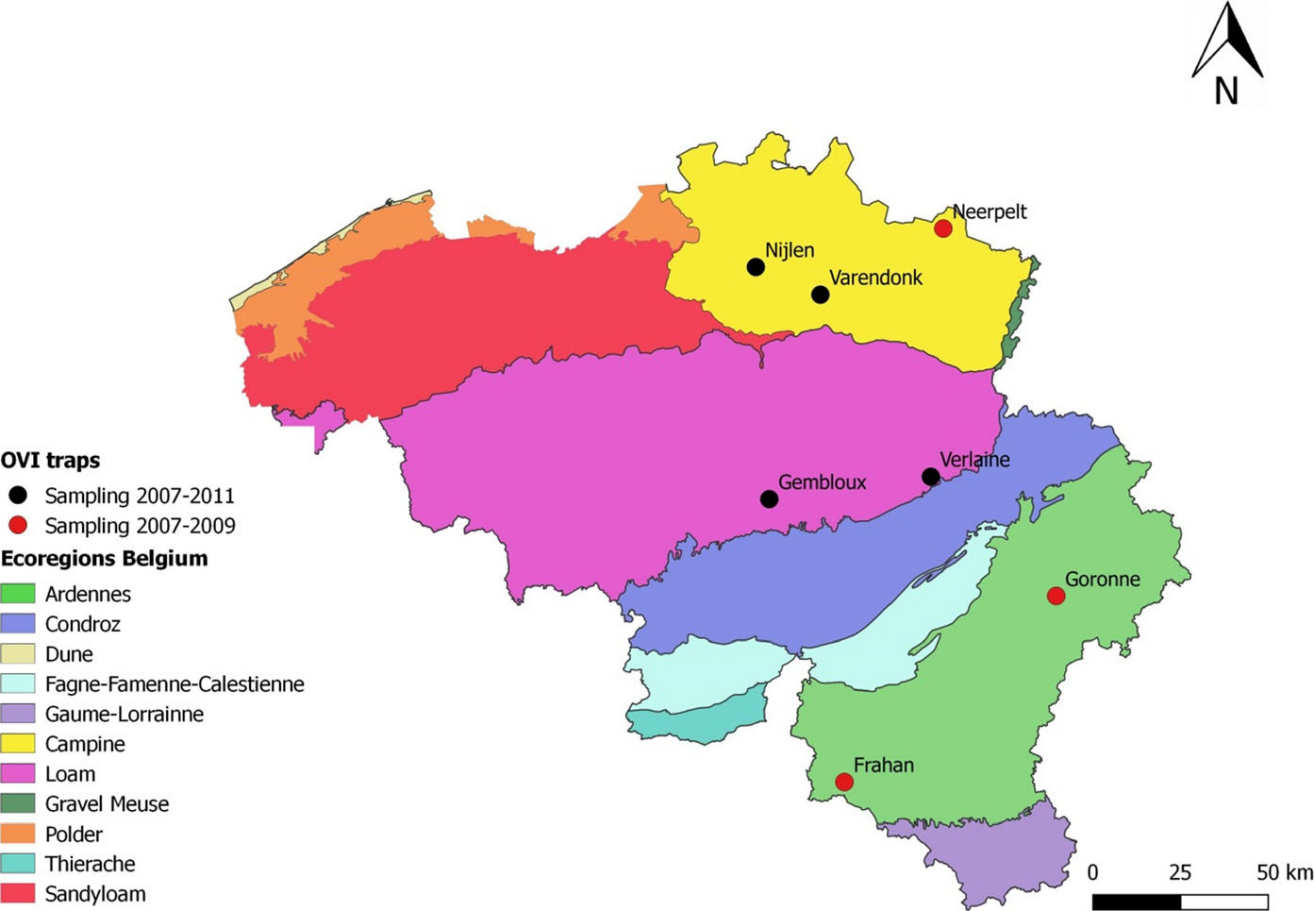
*Culicoides* trapping locations in Belgium with eco-regions

*Culicoides* were captured on seven animal farms in Belgium (Additional file 1: Table S1). The collection sites were distributed over the Flemish (Nijlen, Varendonk and Neerpelt) and Walloon (Frahan, Goronne, Verlaine and Gembloux) administrative parts of Belgium (Fig. 1). Three farms were monitored for three years (April 2007-December 2009) and four other farms were monitored for five consecutive years (April 2007-December 2011) (Fig. 1). Dairy farms with at least 20 cows and located 10 km apart were selected and were distributed over different eco-regionsin Belgium (Additional file 1: Table S1, Fig. 1). All farmers had to give the permission to place traps in their farms. *Culicoides* midges were collected with OVI traps (12 V; 8 Watt; Onderstepoort, SA) installed outside at 1.5–2.0 m above ground level immediately next to the stable or in a tree within < 30 m of the stable in close proximity to animals. Only in Goronne the trap was actually hung in a meadow further away [< 200 m of the stable where animals were present except in winter (Additional file 1: Table S1)]. Traps were placed outdoors on exactly the same trapping location at the different sites throughout the entire study and operated one night on a weekly or biweekly basis. At the beginning and end of the vector season, *Culicoide*s were collected weekly at all sites.

Insects were collected and stored in 70% alcohol in the laboratory until the moment of analysis. In a first step, captured *Culicoides* were separated from other insects using the characteristic features of wings, antennae and legs and the number of *Culicoides* in each collection was counted. In a second step, *Culicoides* were identified up to complex or species level using the key of Delécolle [9] and sorted according to sex. If collections exceeded 200 *Culicoides*, a randomly selected subsample of 200 midges was identified, and numbers of identified species were extrapolated to the total number of midges obtained in that collection.

### Abundance

Three parameters were used to describe abundance. The total yearly abundance represents the total number of collected *Culicoides* at a site. In order to be able to compare this parameter between sites, only data from collection time points at which the traps had operated at all sites were used . Secondly, the maximum annual abundance was determined at each site, representing the most abundant collection obtained during 1 night. Thirdly, the monthly average abundance was determined at all individual sites and over all sites together.

### Vector-free period

Since *C. imicola* is absent in Belgium and the parity status was not determined for all collections, we considered the vector-free period at each collection site as the period between the latest date at which 5 female *Culicoides* were collected during a 1 night collection period at the end of a season and the first date that again 5 female *Culicoides* were collected during the next season.

### Statistical analysis

To determine whether certain collection sites allowed constitutively more abundant collections or defining longer vector-free periods, a ranking was made for each year where the site with the most abundant collection or longest vector-free period, respectively, received the highest rank and the average rank was calculated. Friedman’s test was used to determine whether significant differences between ranks were present. If significant differences were found, two-by-two comparisons of locations were carried out using Wilcoxon *post-hoc* tests. A Bonferroni correction to compensate for the number of two-by-two comparisons was applied.

Statistical analyses were done using SPPS statistics 23. *P* values < 0.05 were considered to be significant. QGIS software was used to produce the map of Belgium.

## Results

### Species composition

During the observational period 2007–2011 a total of 363,115 *Culicoides* were collected at the seven sites. Forty-two different species were morphologically identified. *C. obsoletus* species complex comprising *C. obsoletus* (*s.s.*) and *C. scoticus* accounted together for 70% of all individuals. Other prominent species were *C. dewulfi* (9%) and *C. chiopterus* (4%), meaning the subgenus *Avaritia* covered 83% of all collected individuals. *Culicoides punctatus* (5%), *C. pulicaris* (4%), *C. kibunensis* (3%), *C. archayi* (1%) and *C. festivipennis* (1%) were also abundant. The other 33 species accounted together for about 3% of the collection. No *C. imicola* were collected at any of the sites.

Despite the overall dominance of *Avaritia* species, important variations in species composition were found between sites, and even between different years at the same site (Additional file 2: Table S2). For example, the relative abundance of *C. obsoletus* complex midges ranged between 60–90% at most sites, while it was only 31% in Verlaine. At that location, depending on the year, either *C. achrayi*, *C. kibunensis* or *C. pulicaris* had high relative abundances (Additional file 2: Table S2). At Varendonk, the relative abundance of the *C. obsoletus* complex ranged between 70–80% in 2007, 2008 and 2011 while it dropped to 40–50% and was replaced by increasing relative abundances of *C. dewulfi* in 2009 and *C. dewulfi* and *C. chiopterus* in 2010. Furthermore, some species that were absent or present in low numbers at most locations were highly abundant at other locations, e.g. the relative abundance of 44% of *C. achrayi* in 2007 and of 49% of *C. kibunensis* in 2010 at Verlaine while these species had only a low abundance at other locations.

### Total yearly abundances

To be able to compare total abundances found at the seven sites, only data from collection time points that traps operated at all sites during a specific year were used. Data in Table 1 show that each year, considerable differences in total abundances between sites were found, with up to 16-fold difference between sites with the highest and lowest total abundance. Furthermore, the lowest total yearly abundance was found four times at Nijlen while the highest total yearly abundance was three times obtained at Gembloux and twice at Frahan. This indicates that some sites consistently allow higher or lower total annual abundances and this was statistically confirmed by Friedman’s tests (2007–2009, 7 sites, *χ*^2^ = 14.00, *df* = 6, *P* = 0.030; 2007–2011, 4 sites, *χ*^2^ = 12.840, *df* = 3, *P* = 0.005). Further two-by-two comparisons of sites with Wilcoxon tests did not reveal significant differences between sites after applying the Bonferroni correction, most probably caused by the limited number of repetitions (3 or 5).

**Table 1:** Total yearly abundances at comparable collection time points at each location between 2007–2011

Site	2007 (*n* = 29)	2008 (*n* = 24)	2009 (*n* = 22)	2010 (*n* = 25)	2011 (*n* = 38)	Average inter-site rank 2007–2009; 7 sites	Average inter-site rank 2007–2011; 4 sites
Nijlen	696	8269	1020	1015	1824	1.67	1.20
Varendonk	3634	14,658	2586	5151	14,992	4.33	2.80
Neerpelt	1558	2242	3809	–	–	2.67	–
Frahan	10,277	12,135	9569	–	–	6.33	–
Goronne	8702	11,773	6233	–	–	5.00	–
Verlaine	1111	2549	1139	7652	7610	2.00	2.00
Gembloux	9060	17,957	6208	16,476	20,319	6.00	4.00
Fold difference ^a^	15	8	9	16	11	–	–

*Abbreviation*: *n* number of comparable collection time points

^a^Fold difference between sites with the highest and lowest total yearly abundance

### Maximum annual abundances

The maximum number of midges collected during one collection ranged from 336 at Nijlen (June 2011) to 25,445 at Gembloux (May 2008). Depending on the year, a 10- to 30-fold difference was found between the sites with the highest and lowest maximum annual abundance. For 3 consecutive years, 2008 to 2010, the highest maximum annual abundance was found at the collection site in Gembloux. In 2007 and 2011 the highest maximum yearly abundance was found in Goronne and Varendonk, respectively (Table 2). On the other hand, the lowest maximum annual abundance was found each year at Verlaine or at Nijlen. The observation that some sites constantly showed a higher or lower maximum annual abundance was statistically confirmed for the 5-year monitoring at 4 sites (*χ*^2^ = 10.920, *df* = 3, *P* = 0.012) and was almost significant for the monitoring of 7 sites for 3 years (*χ*^2^ = 12.00, *df* = 6, *P* = 0.062) using Friedman’s ranking tests.

**Table 2:** Maximum annual abundance and capture date at 7 collection sites in Belgium

Site	2007	2008	2009	2010	2011	Average inter-site rank 2007–2009; 7 sites	Average inter-site rank 2007–2011; 4 sites
Nijlen	1186 (19/06/2007)	3289 (29/07/2008)	457 (11/08/2009)	471 (29/06/2010)	336 (28/06/2011)	2.33	1.4
Varendonk	2337 (9/05/2007)	12,000 (22/04/2008)	1829 (30/06/2009)	1859 (15/06/2010)	8787 (12/10/2011)	4.67	3
Neerpelt	708 (19/06/2007)	4700 (8/07/2008)	3462 (30/06/2009)	-	-	3.67	-
Frahan	1941 (13/09/2007)	8310 (6/05/2008)	5046 (1/07/2009)	-	-	5	-
Goronne	13,896 (2/10/2007)	2450 (11/08/2008)	3005 (19/05/2009)	-	-	4.33	-
Verlaine	495 (6/06/2007)	791 (27/05/2008)	1044 (16/07/2009)	2166 (29/06/2010)	1699 (30/05/2011)	1.33	1.8
Gembloux	4000 (9/10/2007)	25,445 (14/05/2008)	5200 (9/06/2009)	10,935 (30/06/2010)	6700 (23/08/2011)	6.67	3.8
Fold difference^a^	28	32	11	23	26	-	-

^a^Fold difference between sites with the highest and lowest maximum annual abundance

Furthermore, it was observed that huge variation existed in the moment at which the maximum annual abundance was found, both between the sites during a specific year (e.g. May to October in 2007 and 2011) and between years at a specific site (e.g. April to October in Varendonk) (Table 2).

### Mean monthly abundance

**Fig. 2:**
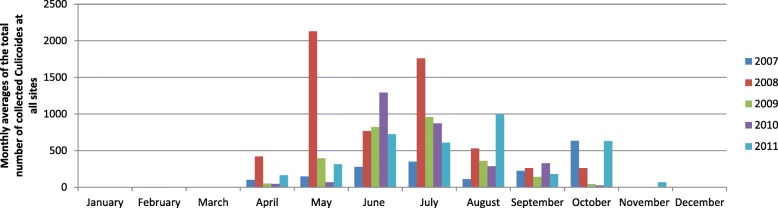
Monthly averages of the number of *Culicoides* collected over all sites between 2007–2011

Figure 2 shows the average number of *Culicoides* collected per month over all sites for the different years the monitoring was performed. It shows that very low or no *Culicoides* were collected between November and March, while most *Culicoides* were collected during June, July and August. However, the month in which most *Culicoides* were collected on average varied greatly over the years, ranging from May in 2008, June in 2010, July in 2009, August in 2011 and October in 2007. Even more variation in the month in which most *Culicoides* were collected on average was observed when looking at individual collection sites. For example in 2011, the highest monthly average was found in May at Verlaine while it was only in October at Varendonk. It was furthermore not constant over the years since for example at Varendonk, the highest monthly average was found in April in 2008 while it was only in October in 2011 (Table 3).

**Table 3:** Month with the highest monthly average of *Culicoides* collected at each site from 2007–2011

Site	2007	2008	2009	2010	2011
Nijlen	June	July	August	July	June
Varendonk	May	April	June	June	October
Neerpelt	July	July	June	–	–
Frahan	September	May	July	–	–
Goronne	October	August	May	–	–
Verlaine	June	May	July	June	May
Gembloux	October	May	June	June	August

### Vector-free period

Considering data from all collection sites, the start of the vector-free period, and thus the last yearly collection of five female midges, ranged approximately from mid-October to mid-November. The end of the vector-free period, and thus the first yearly collection of five female midges occurred on average in early April, with the first record in late March (Table 4). Members of the *Obsoletus* complex were always among the first midges collected at each site. Depending on the year and the collection site, the vector-free period ranged between 127 and 211 days and was 173 days on average (Table 4).

**Table 4:** Duration in days and dates of the seasonally vector-free periods at the 7 collection sites

	2007/2008	2008/2009	2009/2010	2010/2011	2011/2012
Nijlen	197 (09.10–22.04)	127 (25.11–31.03)	201 (12.11–31.05)	211 (21.09–19.04)	147 (08.11–03.04)
Varendonk	147 (20.11–15.04)	140 (12.11–31.03)	183 (06.10–06.04)	163 (08.11–19.04)	140 (08.11–27.03)
Neerpelt	190 (16.10–22.04)	199 (07.10–23.04)	– (06.10)	–	–
Frahan	154 (20.11–22.04)	183 (21.10–21.04)	– (17.11)	–	–
Goronne	183 (29.10–28.04)	190 (21.10–28.04)	– (10.11)	–	–
Verlaine	177 (29.10–22.04)	198 (06.10–21.04)	197 (17.11–01.06)	154 (03.11–05.04)	166 (28.10–11.04)
Gembloux	170 (16.10–02.04)	176 (21.10–14.04)	183 (27.10–27.04)	168 (20.10–05.04)	141 (08.11–28.03)
Difference in days^a^	50	72	18	57	26

^a^Difference in days between sites with the longest and shortest vector free period

Importantly, within a given year, differences in the length of the vector-free period of about 1–2 months were found between different collection sites, showing that the duration of the vector-free period can strongly be influenced by the selection of the collection site. In contrast to the abundance parameters, however, no sites could be identified where consistently shorter or longer vector-free periods were found (Friedman’s tests: 2007–2009, 7 sites, *χ*^2^ = 7.071, *df* = 6 , *P* = 0.314; 2007–2011, 4 sites, *χ*^2^ = 5.449 , *df* = 3, *P* = 0.142).

## Discussion

In this study, we describe the results of a 5-year longitudinal *Culicoides* monitoring in Belgium, with as main goal to analyze whether variation in abundance and seasonality found between collection sites during a one year monitoring of 2011 [3] was consistent over multiple years and to evaluate the consequences thereof for risk assessment on spread of *Culicoides*-borne diseases and determination of vector-free periods.

During the monitoring, a total of 42 different species of the genus *Culicoides* were identified. *Culicoides* belonging to the subgenus *Avaritia* (*C. obsoletus*/*scoticus*, *C. dewulfi* and *C. chiopterus*) together with *C. punctatus* and *C. pulicaris* (subgenus *Culicoides*), which comprise the most important putative vectors of BTV and SBV, were the most abundant and widely distributed species in Belgium. They were present for most part of the year (data not shown). Other species are present, but in much lower quantities and sometimes for a more limited period of the year. Due to the use of OVI light traps for the collections, some diurnal species (e.g. *C. vexans*, *C. nubeculosus* and *C. pulicaris* [10–12]) might have been missed or underestimated since these are not usually captured by light traps or are less attracted by UV light (e.g. *C. chiopterus* [13]). Overall, the *Culicoides* fauna observed in this longitudinal monitoring from 2007 to 2011 is in line with what was previously reported for the *Culicoides* monitoring in 2011 in Belgium [3].

Our analysis of abundance parameters shows that there is important yearly variation in *Culicoides* abundance between collection sites distributed over Belgium and that this yearly variation is consistent over several years. Some sites consistently allowed collecting higher or lower total and maximum annual abundances. This is in line with the outcome of other longitudinal monitoring studies of *Culicoides*. In Switzerland, important differences in abundance were found between 12 traps covering the different climatic regions of the country, but only low yearly variation in abundance was found at each location over a three year monitoring period [14]. Although we did not statistically compare the abundance at specific sites over multiple years, but rather looked at differences between sites at different years of the longitudinal monitoring, our results support the conclusion made by Kaufman et al. [14] that monitoring of midge abundance should preferentially be done by investigating a large number of sites during one season, instead of monitoring a few locations for extended periods of time. Our data show that this approach will allow selecting those sites where most abundant collections can be obtained.

Differences in abundance between collection sites have been reported multiple times before [14–24] and several studies identified site-dependent differences in environmental and ecological factors like soil type, land use, proximity to livestock or other suitable hosts and the presence of appropriate breeding sites [14–18, 25] as drivers for these differences. In our study, no ecological factors in the immediate vicinity of the collection sites or characteristic aspects related to the eco-region of the collection sites could be identified explaining the high abundances at Gembloux and Frahan and low abundances at Nijlen and Verlaine. It is however important to mention that local parameters at the collection site can strongly influence abundance since important differences (up to eight-fold in total yearly abundance and 25-fold in maximum annual abundance) between close by located sites as Nijlen and Varendonk were found. Similar important differences in abundance were reported between two farms only four km apart in Switzerland [21].

Besides variation in abundance between collection sites, also important seasonal variations were observed between sites during a particular year and over the years at individual sites with peaks in abundance varying from May to October. This important variation in seasonality between relatively nearby located collection sites was somewhat unexpected, since seasonality is thought to be mostly driven by climate, and especially temperature and precipitation variables [19, 25]. Since Belgium is a small country that completely belongs to the same climate type (temperate maritime climate; Cfb climate, Köppen-Geiger classification), it seems that generalized meteorological data cannot account for the observed differences and that it are rather local micro-climate environments that strongly impact *Culicoides* seasonality. Such local climate parameters were however not recorded during our monitoring study, so we cannot further elaborate thereon. The observed local variation in seasonality seems however in line with a recent report showing that microclimates can differ strongly at close by locations, even at the same farm [26]. From all this, it can be concluded that a monitoring over several years is necessary when one wants to get profound insights in (the variation in) *Culicoides* seasonality in a region or country.

An important question is whether abundance data obtained during *Culicoides* monitoring campaigns can be used to assess the risk of disease transmission. Recent analyses of *Culicoides* monitoring data over a transect from southern (Spain) to northern (Norway) Europe identified several major spatial patterns and temporal trends of several *Culicoides* species ensembles that were estimated as a relevant overview of transmission potential in Europe to be used for international prevention programmes [27]. Such an approach certainly captures large scale trends, but when looking at a more local (country) scale, our and other results mentioned above indicate that the outcome of vector monitoring programmes can be strongly influenced by the selection of the collection sites. This implicates that extrapolation of abundance parameters like total and maximum abundance should be done carefully and that these should be used cautiously in risk assessments for the spread of *Culicoides*-borne diseases. We found up to 30-fold differences in maximum annual abundance between sites during one year, meaning that the risk of transmission of a *Culicoides*-borne disease could easily be over- or underestimated if not sufficient sites are monitored and that local differences could be difficult to predict. It seems therefore advisable to perform a one-year monitoring of multiple sites to identify sites that allow most abundant collections and use data from those sites to estimate a worst case scenario for disease transmission.

Another pitfall that might complicate risk assessments for spread of *Culicoides*-borne diseases based on abundance parameters is the variation in the period that *Culicoides* are most abundant. Our results show that the moment of maximum abundance varies greatly between years, and that the maximum abundance might occur at moments that there is no risk for disease transmission. For currently unknown reasons, SBV and BTV were detected mostly in midges from August onwards [3, 28–30], suggesting that abundant collections obtained in the beginning of the season might have little importance for disease transmission and might complicate modeling.

Taken together, our data show that acquiring representative abundance and seasonality data asks important monitoring efforts and that even then these should only be cautiously used in risk assessments for spread of vector-borne diseases. Such assessments should furthermore be made by experienced people familiar with all parameters influencing disease transmission, like abundance, seasonality, environmental temperature, wind speed, etc. [3, 18, 31, 32].

It is important to note that risk assessments for transmission of *Culicoides*-borne diseases normally rely on data from female midges of putative vector species, since only these are capable to spread the pathogens, while we used data comprising all species and both sexes in our analysis of abundance and seasonality. The fact that 87% of all collected *Culicoides* in this study are considered to be putative vectors (*C. chiopterus*, *C. dewulfi*, *C. obsoletus*, *C. scoticus* and *C. pulicaris*) [30, 33, 34] and that only 6.3% were males makes us confident that our conclusions regarding the use of abundance data for risk assessments are valid and that an analysis with only female putative vector species would lead to a similar outcome.

One of the most important purposes of installing *Culicoides* monitoring programmes in several European countries after the BTV-8 outbreak of 2006 was to define the vector-free period to enable safe movements of susceptible livestock [1, 3]. The legislative instructions on how this monitoring should be organized are however minimal and leave many aspects open to the interpretation of the participating member states. Our results and those of others on variation in abundance and vector-free period [14–24, 27] between collections sites however argue that it would be advisable to provide more strict guidelines on the number of collection sites to monitor per surface area or per climatic- or eco-region, maybe after an initial screening to select collection sites allowing abundant collections. Also basic recommendations on aspects like trap types to use (UV traps) [35], locations to install the trap (outside, in the near vicinity to farm animals, away from other light sources [14], at a trapping height of 1.5–2.0 m) and the length of the collection period (24–48 h) would ascertain that relevant data are gathered to determine the vector-free period.

One of our most striking findings is that within one year, differences of up to two months in vector-free period were found between relatively close by collection sites. Although we have no straightforward explanation for this observation, it further indicates that one should be careful to extrapolate obtained vector-free periods from one site over a large area. Furthermore, our observed variation in vector-free periods between years at specific sites, which mostly coincides with variation at the end of the vector activity period, supports the conclusion of Searle et al. [36] that a continuous monitoring is necessary and cannot be replaced by modeling, and that most efforts should be done to correctly determine the start of the vector-free period.

Other suggestions made before by others should also be taken into account when the idea would be considered to implement more detailed legislative monitoring guidelines to determine the vector-free period. Cuellar et al. [27] recently argued against the use of specific temperature thresholds to define vector-free periods, since they found that midges tend to be capable to be active at lower temperatures at more northern latitudes. Searle et al. [36] suggested that potentially also differences in phenology between species of the *Avaritia* group should be considered in determining the vector-free period, certainly if it would be shown that these have different vector competences. This latter aspect is currently however little studied and understood only to a limited extent.

## Conclusions

Our data highlighting the important variation in *Culicoides* monitoring results depending on the collection site suggest that information from multiple collection sites over several years should be gathered in order to acquire representative abundance and seasonality data and that even then, these parameters should only be cautiously used in risk assessments. The observed variation in vector-free period between individual trapping locations and its impact on decision making suggests that attempts should be made to define more strict criteria for determination of the vector-free period.

## Additional files

Additional file 1: **Table S1.** Location, sampling period, local ecological factors in the immediate vicinity of the collection site and eco-region with its characteristic ecological aspects for all 7 collection sites. (DOCX 14 kb)

Additional file 2: **Table S2.** Species diversity and relative abundance (%) of *Culicoides* collected with OVI traps from 2007 to 2011 at 7 sites in Belgium). (DOCX 28 kb)

## Abbreviations

EFSA: European Food Safety Authority
ECDC: European Centre for Disease Prevention and Control
OVI traps: Onderstepoort Veterinary Institute blacklight traps
BTV: Bluetongue virus
SBV: Schmallenberg virus
