# Supplementary material for: Longitudinal monitoring of Culicoides in Belgium between 2007 and 2011: local variation in population dynamics parameters warrant cautious use of monitoring data
Source: Parasit Vectors. 2018 Sep 17;11:512. doi: 10.1186/s13071-018-3082-3 (PMC6142705; doi:10.1186/s13071-018-3082-3)
Supplement: Supplementary file 1 — Table S1. Location, sampling period, local ecological factors in the immediate vicinity of the collection site and eco-region with its characteristic ecological aspects for all 7 collection sites. (DOCX 14 kb) [file 13071_2018_3082_MOESM1_ESM.docx]

Additional file 1: Table S1. Location, sampling period, local ecological factors in the immediate vicinity of the collection site and ecoregion with its characteristic ecological aspects for all 7 collection sites

| **Location** | **Province** | **Longitude** | **Latitude** | **Sampling period** | **Trap position** | **Livestock** | **Forest in immediate vicinity** | **Water in immediate vicinity** | **Eco-region** | **Soil type** | **Main vegetation** |
| --- | --- | --- | --- | --- | --- | --- | --- | --- | --- | --- | --- |
| Nijlen | Antwerp | 4.693747 | 51.159744 | 2007-2011 | Stable | Dairy cows | no | no | Campines | sand | heathland |
| Varendonk | Antwerp | 4.954160 | 51.085820 | 2007-2011 | Stable | Dairy cows | yes | pond | Campines | sand | heathland |
| Neerpelt | Limburg | 5.463570 | 51.244950 | 2007-2009 | In tree <30m from stable | Dairy cows | yes | small pond | Campines | sand | heathland |
| Frahan | Luxembourg | 4.998744 | 49.834608 | 2007-2009 | In tree <30m from stable | Dairy cows/Pony’s | yes | river | Ardennes | sandstone & slate | forest |
| Goronne | Luxembourg | 5.866575 | 50.293831 | 2007-2009 | In tree <200m from stable | Dairy cows | yes | no | Ardennes | sandstone & slate | forest |
| Verlaine | Liège | 5.379914 | 50.611208 | 2007-2011 | In tree <30m from stable | Dairy cows | yes | small river | Loam | loam | arable crops |
| Gembloux | Namur | 4.726620 | 50.565090 | 2007-2011 | Stable | Dairy cows/pigs/horses | no | no | Loam | loam | arable crops |
