# Supplementary material for: Longitudinal monitoring of Culicoides in Belgium between 2007 and 2011: local variation in population dynamics parameters warrant cautious use of monitoring data
Source: Parasit Vectors. 2018 Sep 17;11:512. doi: 10.1186/s13071-018-3082-3 (PMC6142705; doi:10.1186/s13071-018-3082-3)
Supplement: Supplementary file 2 — Table S2. Species diversity and relative abundance (%) of Culicoides collected with OVI traps from 2007 to 2011 at 7 sites in Belgium). (DOCX 28 kb) [file 13071_2018_3082_MOESM2_ESM.docx]

Additional file 2: Table S2. Species diversity and relative abundance (%) of *Culicoides* collected with OVI traps from 2007 to 2011 at 7 sites in Belgium.

| **Subgenus** | **Species** | **Nijlen** | | | | | | **Varendonk** | | | | | | **Neerpelt** | | | | **Frahan** | | | | **Goronne** | | | | **Verlaine** | | | | | | **Gembloux** | | | | | **Tot** |
| --- | --- | --- | --- | --- | --- | --- | --- | --- | --- | --- | --- | --- | --- | --- | --- | --- | --- | --- | --- | --- | --- | --- | --- | --- | --- | --- | --- | --- | --- | --- | --- | --- | --- | --- | --- | --- | --- |
|  |  | 7 | 8 | 9 | 10 | 11 | **Tot** | 7 | 8 | 9 | 10 | 11 | **Tot** | 7 | 8 | 9 | **Tot** | 7 | 8 | 9 | **Tot** | 7 | 8 | 9 | **Tot** | 7 | 8 | 9 | 10 | 11 | **Tot** | 7 | 8 | 10 | 11 | **Tot** |  |
| *Avaritia* | *C chiopterus* | 6 | 2 | 2 | 6 | 4 | **3** | 14 | 1 | 7 | 15 | 7 | **6** | 5 | 2 | 2 | **2** | 2 | 1 | 2 | **1** | 5 | 4 | 1 | **4** | 3 | 2 | 2 | 1 | 1 | **1** | 3 | 4 | 4 | 3 | **4** | **4** |
|  | *C dewulfi* | 23 | 24 | 10 | 7 | 5 | **20** | 7 | 10 | 44 | 18 | 12 | **13** | 12 | 24 | 14 | **19** | 3 | 3 | 5 | **3** | 6 | 16 | 27 | **14** | 6 | 5 | 11 | 1 | 5 | **4** | 7 | 12 | 2 | 4 | **8** | **9** |
|  | *C obsoletus s.l.* | 66 | 64 | 73 | 79 | 78 | **67** | 72 | 71 | 39 | 50 | 78 | **69** | 71 | 65 | 65 | **66** | 91 | 93 | 87 | **91** | 76 | 45 | 37 | ***59*** | *36* | *36* | *36* | *20* | *38* | ***31*** | 76 | 66 | 79 | 80 | **72** | **70** |
| *Beltranmyia* | *C circumscriptus* | 0 | 2 | 1 | 1 | 0 | **2** | 0 | 0 | 1 | 0 | 0 | **0** | 0 | 0 | 2 | **1** | 0 | 0 | 0 | **0** | 0 | 0 | 0 | **0** | 0 | 0 | 0 | 0 | 0 | **0** | 0 | 1 | 0 | 0 | **0** | **0** |
| *Culicoides* | *C deltus* | 0 | 0 | 0 | 0 | 0 | **0** | 0 | 0 | 0 | 0 | 0 | **0** | 0 | 0 | 0 | **0** | 0 | 0 | 0 | **0** | 1 | 3 | 0 | **1** | 0 | 0 | 0 | 0 | 0 | **0** | 0 | 0 | 0 | 0 | **0** | **0** |
|  | *C impunctatus* | 0 | 0 | 0 | 0 | 0 | **0** | 0 | 0 | 0 | 0 | 0 | **0** | 0 | 0 | 0 | **0** | 0 | 0 | 0 | **0** | 0 | 0 | 7 | **2** | 0 | 0 | 0 | 0 | 0 | **0** | 0 | 0 | 0 | 0 | **0** | **0** |
|  | *C lupicaris* | 0 | 0 | 0 | 0 | 0 | **0** | 0 | 0 | 0 | 0 | 0 | **0** | 0 | 0 | 0 | **0** | 0 | 0 | 0 | **0** | 2 | 0 | 0 | **1** | 0 | 0 | 0 | 0 | 0 | **0** | 0 | 0 | 0 | 0 | **0** | **0** |
|  | *C pulicaris* | 1 | 1 | 1 | 1 | 1 | **1** | 1 | 2 | 1 | 1 | 1 | **1** | 2 | 2 | 2 | **2** | 2 | 2 | 2 | **2** | 3 | 9 | 4 | **5** | 1 | 22 | 3 | 2 | 10 | **7** | 5 | 8 | 3 | 7 | **6** | **4** |
|  | *C punctatus* | 3 | 5 | 2 | 1 | 10 | **5** | 3 | 14 | 3 | 6 | 2 | **8** | 6 | 4 | 7 | **5** | 1 | 1 | 2 | **1** | 2 | 3 | 1 | **2** | 2 | 4 | 1 | 2 | 2 | **2** | 8 | 7 | 2 | 1 | **6** | **5** |
| *Monoculicoides* | *C nubeculosus* | 0 | 1 | 5 | 0 | 0 | **1** | 0 | 0 | 2 | 0 | 0 | **0** | 0 | 0 | 0 | **0** | 0 | 0 | 0 | **0** | 0 | 0 | 0 | **0** | 0 | 0 | 0 | 0 | 0 | **0** | 0 | 1 | 0 | 0 | **0** | **0** |
|  | *C riethi* | 0 | 1 | 4 | 2 | 0 | **1** | 0 | 0 | 2 | 0 | 0 | **0** | 0 | 0 | 0 | **0** | 0 | 0 | 0 | **0** | 0 | 0 | 0 | **0** | 0 | 0 | 0 | 0 | 0 | **0** | 0 | 0 | 0 | 0 | **0** | **0** |
| *Oecacta* | *C festivipennis* | 0 | 0 | 0 | 0 | 0 | **0** | 2 | 0 | 0 | 1 | 0 | **1** | 1 | 1 | 1 | **1** | 0 | 0 | 0 | **0** | 0 | 4 | 0 | **1** | 0 | 2 | 2 | 11 | 4 | **6** | 0 | 0 | 1 | 0 | **0** | **1** |
|  | *C furcillatus* | 0 | 0 | 0 | 0 | 0 | **0** | 0 | 0 | 0 | 0 | 0 | **0** | 0 | 0 | 0 | **0** | 0 | 0 | 0 | **0** | 0 | 0 | 0 | **0** | 0 | 0 | 0 | 1 | 0 | **1** | 0 | 0 | 0 | 0 | **0** | **0** |
|  | *C kibunensis* | 0 | 0 | 0 | 1 | 0 | **0** | 0 | 0 | 1 | 4 | 0 | **0** | 3 | 1 | 6 | **3** | 0 | 0 | 0 | **0** | 0 | 4 | 10 | **3** | 6 | 8 | 40 | 49 | 31 | **35** | 0 | 0 | 5 | 1 | **1** | **3** |
|  | *C pictipennis* | 0 | 0 | 0 | 0 | 0 | **0** | 0 | 0 | 0 | 0 | 0 | **0** | 0 | 0 | 0 | **0** | 0 | 0 | 0 | **0** | 0 | 0 | 2 | **1** | 2 | 3 | 2 | 2 | 4 | **2** | 0 | 0 | 0 | 0 | **0** | **0** |
|  | *C poperinghensis* | 0 | 0 | 0 | 0 | 0 | **0** | 0 | 0 | 0 | 0 | 0 | **0** | 0 | 0 | 0 | **0** | 0 | 0 | 0 | **0** | 0 | 0 | 0 | **0** | 0 | 0 | 0 | 1 | 1 | **1** | 0 | 0 | 0 | 0 | **0** | **0** |
| *Silvaticulioides* | *C achrayi* | 0 | 0 | 0 | 0 | 0 | **0** | 1 | 0 | 0 | 2 | 0 | **0** | 0 | 0 | 0 | **0** | 0 | 0 | 1 | **1** | 3 | 11 | 1 | **5** | 44 | 17 | 2 | 6 | 2 | **8** | 0 | 0 | 2 | 0 | **0** | **1** |
|  | *C subfasciipennis* | 0 | 0 | 0 | 0 | 0 | **0** | 0 | 0 | 0 | 0 | 0 | **0** | 0 | 0 | 0 | **0** | 0 | 0 | 0 | **0** | 0 | 0 | 0 | **0** | 0 | 0 | 0 | 3 | 1 | **1** | 0 | 0 | 0 | 0 | **0** | **0** |
| *Wirthomyia* | *C segnis* | 0 | 0 | 0 | 0 | 0 | **0** | 0 | 0 | 0 | 0 | 0 | **0** | 0 | 0 | 0 | **0** | 0 | 0 | 0 | **0** | 0 | 0 | 8 | **2** | 0 | 0 | 0 | 0 | 0 | **0** | 0 | 0 | 0 | 0 | **0** | **0** |
| *Others* |  | 1 | 1 | 0 | 0 | 1 | **1** | 1 | 1 | 0 | 2 | 0 | **1** | 0 | 0 | 1 | **0** | 0 | 0 | 0 | **0** | 0 | 0 | 1 | **0** | 0 | 0 | 0 | 2 | 2 | **1** | 1 | 0 | 2 | 4 | **1** | **1** |
|  | # *species* | 16 | 23 | 15 | 14 | 22 | **28** | 14 | 18 | 15 | 24 | 22 | **32** | 18 | 12 | 18 | **21** | 16 | 17 | 17 | **23** | 15 | 17 | 19 | **22** | 13 | 15 | 15 | 33 | 28 | **35** | 18 | 15 | 25 | 23 | **29** | **42** |

**Tot = Total. *C obsoletus s.l.* consists out of 2 species, namely *C. obsoletus* and *C. scoticus*. Species comprising less than 1 % of the collection are grouped within others. Others = *C. alazanicus, C. albicans, C. albihalteratus, C. brunnicans, C. clastrieri, C. duddingstoni, C. fagineus, C. fascipennis, C. grisescens, C. heliophilus, C. manchuriensis, C. minutissimus, C. newsteadi, C. pallidicornis, C. parroti, C picturatus, C. puncticollis, C. riouxi, C. salinarius, C. simulator, C. stigma, C. vexans.***
